# Registry-based randomised controlled trials: conduct, advantages and challenges—a systematic review

**DOI:** 10.1186/s13063-024-08209-3

**Published:** 2024-06-11

**Authors:** Frances Shiely, Niamh O Shea, Ellen Murphy, Joseph Eustace

**Affiliations:** 1 Trials Research and Methodologies Unit, HRB Clinical Research Facility, University College Cork, 4th Floor Western Gateway Building, Western Road, Cork, Ireland; 2 School of Public Health, University College Cork, 4th Floor Western Gateway Building, Western Road, Cork, Ireland; 3 Health Research Board, Trials Methodology Research Network, University College Cork, Cork, Ireland; 4 Department of Renal Medicine, Cork University Hospital, Cork, Ireland

**Keywords:** Trials methodology, Registry-based randomised controlled trials

## Abstract

**Background:**

Registry-based randomised controlled trials (rRCTs) have been described as pragmatic studies utilising patient data embedded in large-scale registries to facilitate key clinical trial procedures including recruitment, randomisation and the collection of outcome data. Whilst the practice of utilising registries to support the conduct of randomised trials is increasing, the use of the registries within rRCTs is inconsistent. The purpose of this systematic review is to explore the conduct of rRCTs using a patient registry to facilitate trial recruitment and the collection of outcome data, and to discuss the advantages and challenges of rRCTs.

**Methods:**

A systematic search of the literature was conducted using five databases from inception to June 2020: PubMed, Embase (through Ovid), CINAHL, Scopus and the Cochrane Controlled Register of Trials (CENTRAL). The search strategy comprised of MESH terms and key words related to rRCTs. Study selection was performed independently by two reviewers. A risk of bias for each study was completed. A narrative synthesis was conducted.

**Results:**

A total 47,862 titles were screened and 24 rRCTs were included. Eleven rRCTs (45.8%) used more than one registry to facilitate trial conduct. Six rRCTs (25%) randomised participants via a specific randomisation module embedded within a registry. Recruitment ranged between 209 to 106,000 participants. Advantages of rRCTs are recruitment efficiency, shorter trial times, cost effectiveness, outcome data completeness, smaller carbon footprint, lower participant burden and the ability to conduct multiple trials from the same registry. Challenges are data collection/management, quality assurance issues and the timing of informed consent.

**Conclusions:**

Optimising the design of rRCTs is dependent on the capabilities of the registry. New registries should be designed and existing registries reviewed to enable the conduct of rRCTs. At all times, data management and quality assurance of all registry data should be given key consideration. We suggest the inclusion of the term ‘registry-based’ in the title of all rRCT manuscripts and a clear simple breakdown of the registry-based conduct of the trial in the abstract to facilitate indexing in the major databases.

**Supplementary Information:**

The online version contains supplementary material available at 10.1186/s13063-024-08209-3.

## Background

Randomised controlled trials (RCT) remain the gold standard within clinical research for testing the efficacy of new treatments and improving clinical care [1]. However, RCTs are a complex and costly undertaking, often limited by the difficulty in identifying participants, in efficiently randomising them and in maximising their follow-up [2]. The last decade has seen the development of a variation to the traditional RCT design, in the form of registry-based randomised controlled trials (rRCTs) [3–5]. rRCTs are described as trials with a high level of pragmatism utilising patient data embedded in large-scale registries to facilitate a range of clinical trial procedures including, recruitment, randomisation and collection of outcome data [6, 7]. The advantages of rRCTs are potentially substantial and include cost-effectiveness, trial efficiency, a simplified approach to participant enrolment and high participant follow-up rates [6, 8].

Variations to the traditional RCT design have been explored and include ‘Randomised Database Studies’ [9] (the use of both observational methods (routine clinical practice) and experimental methods in addition to the application of randomisation to the data systematically collected in clinical practice), ‘Point of Care Trials’ [10] (an operational approach to conducting clinical trials that integrates clinical research and routine care delivery making trial more accessible to broader and more diverse populations) and ‘Trials within Cohorts’ (TwiCs) [11] (a single cohort infrastructure which enables participants to be identified and outcomes obtained for multiple trials). Registry-based RCTs combine the strengths of these trial methodologies, e.g. access to larger more diverse groups of trial participants, collection of data needed for the trial as part of routine clinical practice; however, the application of registries within rRCTs is yet to be standardised. Mathes et al. [12] examined the features of rRCTs and concluded that there was a need for a checklist to ensure comprehensive reporting for rRCTs. The 2021 published CONSORT extension for the reporting of randomised controlled trials conducted using cohorts and routinely collected data (CONSORT-ROUTINE) has provided this much needed clarity for the reporting of RCTs using a registry [13]. A later study by Karanatsios et al. [14] cited a need to establish universally accepted criteria for the classification of rRCTs. This arises because the application of registries within rRCTs remains inconsistent. For some, the registry is used for just one purpose, perhaps identifying an outcome [15] or the identification of potential participants [16]. For others, the registry has multiple uses and facilitates a combination of trial processes including, participant recruitment, outcome data collection, and in some cases randomisation [3, 17, 18]. A possible definition for rRCTs has been described by Li et al., whereby the registry is used as a platform for participant recruitment and data collection including the acquisition of outcome/endpoint data [7].

Though there is considerable variation on what constitutes a registry [19], for the purposes of our review, we are including trials utilising a patient registry defined as ‘an organized system that uses observational study methods to collect data (clinical and other) to evaluate specified outcomes for a population defined by a particular disease, condition, or exposure, and that serves one or more predetermined scientific, clinical, or policy purposes’ [20]. The purpose of this review is to explore the conduct of rRCTs using a patient registry to facilitate trial recruitment and the collection of outcome data and to discuss the advantages and challenges. This will assist those considering conducting rRCTs embedded within a patient registry to design and implement trials that are efficient, cost-effective, considerate of the environment and useful.

## Methods

### Search strategy

An electronic search of the literature was conducted using the following databases from inception to June 2020: PubMed, Embase (through Ovid), CINAHL, Scopus and the Cochrane Controlled Register of Trials (CENTRAL). A combination of the following MESH terms and key words were used: randomised OR randomized OR randomised controlled trial OR randomized controlled trial (MESH) OR RCT OR ‘randomized clinical trial’ OR pragmatic trial OR randomized database trial OR randomized registry trial OR ‘database study’ AND Registry (MESH) OR ‘registry based’ OR registry based OR register based OR ‘registry trial’ OR rRCT OR register. Search terms were adapted for each database, with English language articles included and no other filters applied. A list of the search strategies for each database is provided in Supplementary file 1. The reference lists of included studies were searched by backward reference and forward citation searching.

### *Inclusion and exclusion* c*riteria*

Inclusion:

- rRCTs utilising a patient registry to facilitate recruitment of participants and at least one outcome measure.
- rRCTs including randomisation at individual or cluster level.

Exclusion:

- Non-randomised or quasi randomised trials.
- rRCT with a published protocol paper but no associated trial paper.

### Study screening and selection

Trials were exported from EndNote X7 to Rayyan QCRI software [21] for title and abstract screening. All titles were reviewed for eligibility by NOS. In the case where uncertainty arose regarding the relevance of a title, abstract screening was conducted independently by two reviewers (NOS and FS). Both NOS and FS then independently screened the full texts of studies considered to be eligible for inclusion. Disagreement was met through consensus with a third reviewer (JE) as required.

### Data extraction and management

Data were extracted on the following: trial title, author and year, disease under investigation, total enrolled, registry name, registry information, role of registry within trial and overall risk of bias. Data extraction was completed by NOS and a double extraction of 10% of the total sample results was completed by EM Trial authors were contacted where additional information or clarification was required.

### *Assessment for* r*isk of bias*

Two reviewers (NOS and EM) independently assessed the risk of bias for each included rRCT using the Cochrane Collaboration’s tool for assessing risk of bias [22]. The risk of bias tool covers six domains of bias: selection bias, performance bias, detection bias, attrition bias, reporting bias and other bias. Following the guidelines for the use of the risk of bias tool, a judgement is made on each domain for each trial. In any case of disagreement, consensus was reached with a third reviewer (FS). Justifications for all risk of bias judgements are also presented.

### *Data* s*ynthesis*

An analysis of the data was conducted based on the Guidance on the Conduct of Narrative Synthesis in Systematic Reviews [23]. Narrative synthesis is a method used in systematic reviews to combine findings from various studies, primarily utilising words and text to summarise and interpret the results. We summarised the general characteristics of each trial and all registry-linked trial activities including recruitment, outcome measurements, randomisation, data collection, quality assurance, cost-effectiveness, study interventions and informed consent.

This systematic review adheres to the PRISMA (Preferred Reporting Items for Systematic Reviews and Meta-Analyses) standardised reporting guidelines to ensure the standardised conduct and reporting of the research [24]. A PRISMA checklist is provided in supplementary file 2.

## Results

### Study selection

**Fig. 1:**
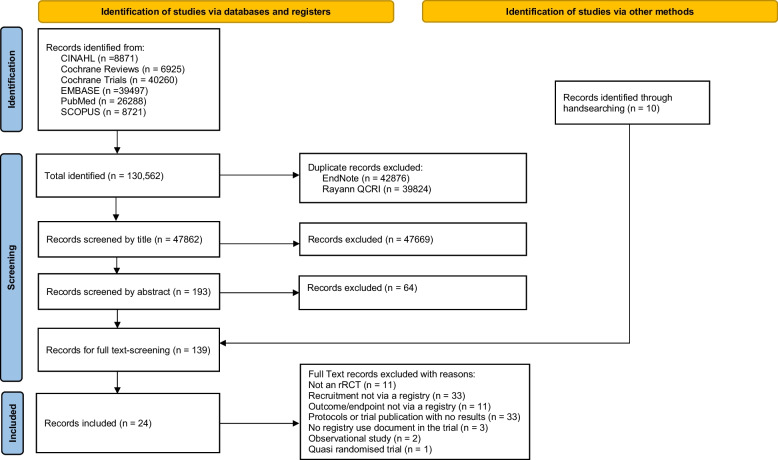
PRISMA flow diagram of the screening process

A total of 130,562 studies were identified and exported to Rayyan QCRI [21]. A search for duplicate studies in EndNote X7 removed 42,876 studies and a second duplicate search in Rayyan QCRI resulted in the removal of an additional 39,824 studies. A total of 47,862 titles were screened for relevance and 193 titles remained for abstract review. Of these, 129 texts underwent full review. An additional ten trials were located from hand searching references. After full text review, a total of 24 trials met the inclusion criteria and were included in the narrative synthesis. The search selection process is detailed in the PRISMA flow diagram (Fig. 1).

### Overview of rRCTs

The characteristics of the rRCTs and their registry-linked activities are presented in Table 1. Publications ranged from 1996 to 2020. The largest number of rRCTs took place in the USA (*n* = 9) followed by the Nordic countries (*n* = 7), Australia (*n* = 2) and the UK (*n* = 2). The remaining rRCTs (*n* = 4) were multiregional. Interventions consisted of drug treatments (*n* = 3) [25–27], surgical procedures (*n* = 2) [8, 28], non-surgical procedures (*n* = 1) [29], disease management (*n* = 3) [30–32], immunisation reminder/recall notices (*n* = 7) [33–39], screening for cancer programmes (*n* = 6) [40–45], methods to improve research participation (*n* = 1) [46] and smoking cessation (*n* = 1) [47]. For five rRCTs (20.8%), the interventions were delivered in a hospital-based setting [8, 25, 27, 28, 48]. Most trials were randomised at the level of the individual participant (*n* = 22) and two (8.3%) were cluster randomised [27, 32]. Recruitment ranged from 209 [30] to 106,000 [43] participants. Eleven rRCTs (45.8%) used more than one registry to facilitate trial procedures [8, 25–29, 40, 42–45].

**Table 1:** rRCT characteristics

Trial title, author, year	Disease under investigation	Total enrolled	Registry name(s)	Registry information	Role of registry within trial	Study findings	Overall risk of bias judgement
1.Effect of Stress Ulcer Prophylaxis with Proton Pump Inhibitors vs Histamine-2 Receptor Blockers on In-Hospital Mortality Among ICU Patients Receiving Invasive Mechanical Ventilation. The PEPTIC Randomized Clinical Trial Young et al. 2020 [1]	Stress ulcer prophylaxis	26,982 participants and 50 ICUs	Australian and New Zealand Intensive Care Society (ANZCIS) Adult Patient Database (APD) Intensive Care National Audit and Research Centre (ICNARC) and Case Mix Programme (CMP) database (UK) eCritical Alberta and eCritical TRACER (Canada) The Irish ICU clinical information Systems (Ireland)	ANZCIS APD—an intensive care database used for routine quality assurance and peer review [2] ICNARC CMP database—a risk prediction model, predicting risk of death before discharge from acute hospitals developed using high-quality clinical databases [3] eCritical Alberta—a clinical information system, eCritical Tracer – a data warehouse and clinical analytics systems [4] Irish ICU clinical information Systems	Patient identification and data collection including primary, secondary and tertiary outcome	Amongst ICU patients requiring mechanical ventilation, a strategy of stress ulcer prophylaxis with use of proton pump inhibitors vs histamine-2 receptor blockers resulted in hospital mortality rates of 18.3% vs 17.5%, respectively, a difference that did not reach the significance threshold. However, study interpretation may be limited by crossover in the use of the assigned medication	High risk
2.Smoking cessation prior to gynecological surgery-A registry-based randomized trial Bohlin et al. 2020 [5]	Preoperative smoking cessation	1427	GynOp Register	Gynop is a Swedish national quality register for gynaecological surgery	Patient recruitment, randomisation (completed within registry), obtaining information about the patient and the surgery performed, and follow up	A combination of written information in the health declaration and a recommendation from a doctor regarding smoking cessation may be associated with higher odds of smoking cessation at 1–3 weeks pre- and postoperatively	Unclear risk
3.Long-Term Effectiveness of Sigmoidoscopy Screening on Colorectal Cancer Incidence and Mortality in Women and Men: A Randomized Trial Holme et al. 2018 [6]	Colorectal cancer	98,678	Population Registry Norway Cancer Registry Norway Population Registry Norway	Population Registry Cancer Registry	Participant identification and trial invitation via population registry, endpoints detection via Cancer Registry, Cause of Death Registry and Population Registry	Offering sigmoidoscopy screening in Norway reduced colorectal cancer incidence and mortality in men, but had little or no effect in women	Low risk
4.Oxygen Therapy in Suspected Acute Myocardial Infarction Hoffmann et al. 2017 [7]	Myocardial infarction	6629	SWEDEHEART Registry Swedish National Inpatient and Outpatient Registries Swedish National Population Registry	SWEDEHEART registry aims to support the improvement of care and evidence-based development of therapy of coronary artery disease [8] Swedish National Inpatient and Outpatient Registries include information on rehospitalization with heart failure and cardiovascular death The Swedish National Population Registry includes the vital status of all Swedish citizens	Patient enrolment, randomisation (via module embedded in SWEDEHEART) and data collection via SWEDEHEART Additional endpoints were obtained from Swedish National Inpatient and Outpatient Registries and Swedish National Population Registry	Routine use of supplemental oxygen in patients with suspected myocardial infarction who did not have hypoxemia was not found to reduce 1-year all-cause mortality	High risk
5.Instantaneous Wave-free Ratio versus Fractional Flow Reserve to Guide PCI Gotberg et al. 2017 [9]	Myocardial infarction	2037	Swedish Coronary Angiography and Angioplasty Registry (SCAAR) The Swedish Web-Based System for Enhancement and Development of Evidence-Based Care in Heart Disease Evaluated According to Recommended Therapies (SWEDEHEART) Registry The Danish National Patient Registry and the Western Denmark Heart Registry	The SCAAR contains information on all patients treated by coronary angiography and PCI in Sweden and Iceland SWEDEHEART is a national registry recording the data of patients suffering myocardial infarction	Participant recruitment (via module embedded within registry), data collection and randomisation via SCAAR End point detection via SWEDEHEART and The Danish National Patient Registry and the Western Denmark Heart Registry	IFR-guided revascularization strategy was noninferior to an FFR-guided revascularization strategy with respect to the rate of major adverse cardiac events at 12 months	High risk
6.Pragmatic Randomized, Controlled Trial of Patient Navigators and Enhanced Personal Health Records in Chronic Kidney Disease Navaneethan et al. 2017 [10]	Chronic kidney disease	209	Chronic Kidney Disease Registry	The registry is an electronic health record-based chronic kidney disease registry	Participant identification, study data collection and outcome measure detection	A patient navigator program and an enhanced personal health record for the CKD population was successfully developed. However, there were no differences in eGFR decline and other outcomes amongst the study groups	Unclear risk
7.Bivalirudin versus Heparin Monotherapy in Myocardial Infarction (Validate Swedeheart trial) Erlinge et al. 2017 [11]	Myocardial infarction	6006	The Swedish Web-system for enhancement and development of evidence-based care in heart disease evaluated according to recommended therapies (SWEDEHEART) Swedish Coronary Angiography and Angioplasty Registry (SCARR) Swedish national population registry	SWEDEHEART registry aims to support the improvement of care and evidence-based development of therapy of coronary artery disease SCAAR is a component of the SWEDEHEART registry	Enrolment and data collection via SWEDEHEART Randomisation (via module embedded within SCARR) Follow up end point detection via pre-existing healthcare registries	The rate of the composite of death from any cause, myocardial infarction, or major bleeding was not lower among those who received bivalirudin than among those who received heparin monotherapy	High risk
8.Home-based HPV self-sampling improves participation by never-screened and under-screened women: Results from a large randomized trial (iPap) in Australia Sultana et al. 2016 [12]	Cervical cancer	8160	Victorian Cervical Cytology Registry (VCCR)	The VCCR records all cervical cytology and associated histology reports for Victorian women and sends reminders to women when their Pap test is overdue	Identification of trial participants and outcome measure detection	Inviting women to self‐sample for HPV testing resulted in a substantially greater participation in screening than an invitation or reminder letter for a Pap test, in both strata of never‐ and under‐screened women	Unclear risk
9.Population-Based Colonoscopy Screening for Colorectal Cancer: A Randomized Clinical Trial Bretthauer et al. 2016 [13]	Colorectal cancer	94,959	Population registries Cancer Registries Registries of Causes of Death	Population registries, Cancer registries and Registries of Causes of Death from Norway, Poland, Sweden, and the Netherlands were used in the trial	Participant identification and recruitment via population registries. The primary outcome was assessed by linkage to cancer registries, population registries and registries of causes of death. patient registries in the participating countries [14]	Colonoscopy screening entails high detection rates in the proximal and distal colon. Participation rates and endoscopist performance vary significantly. Post procedure abdominal pain is common with standard air insufflation and can be significantly reduced by using CO2	Unclear risk
10.Telephone Intervention to Improve Diabetes Control, A Randomized Trial in the New York City A1c Registry Chamany et al. 2015 [15]	Type 2 diabetes	941	A1c Registry	The A1c registry stores the A1c test results for NYC residents. The Department of Health and Mental Hygiene expanded existing community programmes and policy approaches to diabetes prevention and control by creating the A1c Registry	Identification of potential trial participants and outcome measures	A telephone intervention can be an effective tool to improve diabetes control in diverse populations by using a registry	Unclear risk
11.Age-specific strategies for immunization reminders and recalls: a registry-based randomized trial Dombkowski et al. 2014 [16]	Prevention of diphtheria, tetanus, pertussis, hepatitis B, pneumococcal conjugate, polio vaccines, measles, mumps, and rubella, and one varicella	10,175	The Michigan Care Improvement Registry (MCIR), USA	The MCIR is a statewide immunisation information systems database	Identification of participants and collection of outcome data	Although recall notifications can positively affect immunisation activity, the effect may vary by targeted age group. Many 7- and 12-month-olds had immunisation activity following reminder/recall; however, levels of activity were similar irrespective of notification, suggesting that these groups were likely to receive medical care or immunisation services without prompting	Unclear risk
12.Thrombus Aspiration during ST-Segment Elevation Myocardial Infarction TASTE Trial Frobert et al. 2013 [17]	Myocardial infarction	7244	Swedish Coronary Angiography and Angioplasty Registry (SCAAR) National Population Registry The National Discharge Registry	SCAAR contains information on all patients treated by coronary angiography and PCI in Sweden and Iceland. It is part of the internet based SWEDEHEART registry The Population Registry and Discharge Registry are national registries	Participant enrolment, data collection and randomisation via SCAAR. Endpoint detection via national health registries and SWEDEHEART	Routine thrombus aspiration before PCI as compared with PCI alone did not reduce 30-day mortality among patients with STEMI	High risk
13.Format and readability of an enhanced invitation letter did not affect participation rates in a cancer registry-based study: a randomized controlled trial Hall et al. 2012 [18]	Haematological cancer	268	Australian State-based cancer registry	Cancer registry	Participants identified and invited to take part in trial via registry. Data collection via registry Outcome measure of survey response rate recorded in registry	An enhanced invitation letter was not effective in increasing participation of haematological cancer survivors in an Australian cancer registry study	Unclear risk
14.Seasonal influenza vaccination reminders for children with high-risk conditions: a registry-based randomized trial Dombkowski et al. 2012 [19]	Prevention of influenza	3618	Michigan Care Improvement Registry (MCIR), USA	The MCIR is a statewide immunisation information systems database	Identification of participants and collection a main outcome measure (effectiveness analysis)	Receipt of a reminder was positively associated with seasonal influenza vaccination	Unclear risk
15.Test, episode, and programme sensitivities of screening for colorectal cancer as a public health policy in Finland: experimental design Malia et al. 2008 [20]	Colorectal cancer	106,000	Mass Screening Registry Population Register Centre Finnish Cancer Registry	The Mass Screening Registry is a division of the Finnish Cancer Registry The Population Register Centre, holds records including a personal identifier on every Finnish citizen. The identifier enables individual linkage to health registers, such as the cancer registry	The overall design and coordination of the programme was the responsibility of the national Mass Screening Registry (a division of the Finnish Cancer Registry) Participant sampling and invitation via Population Register Centre Follow up was obtained via Finnish Cancer Registry	Although relatively low, the sensitivity of screening for colorectal cancer with the faecal occult blood test in Finland was adequate	Unclear risk
16. A pragmatic cluster randomised controlled trial of a Diabetes REcall And Management system: the DREAM trial Eccles et al. 2007 [21]	Diabetes	58 UK based GP Practices (cluster randomised) and 3608 participants	Diabetes Register	Registry of Diabetes Patients	Recruitment of GP practices (practices had to be linked to the diabetes register to participate) Patient identification and recruitment (patients had to be on the diabetes register to participate) Outcome variables obtained via diabetes registry	This study showed benefits from an area-wide, computerised diabetes register incorporating a full structured recall and individualised patient management system. However, these benefits were achieved at a cost. In future, these costs may fall as electronic data exchange becomes a reliable reality	Unclear risk
17.Challenges and Successes of Immunization Registry Reminders at Inner-City Practices Irigoyen et al. 2006 [22]	Prevention of diphtheria-tetanus-pertussis	1662	EzVAC Registry	EzVAC is a provider-based registry which consolidates immunisation records for a hospital health care system, in New York, USA	EzVAC was programmed to identify eligible children, randomly sample a fraction of those eligible, and then randomly assign those sampled to 1 of 3 study groups The intervention, a registry-based reminder and outcome detection was also facilitated by EzVac	At an inner-city practice network, registry reminders were not effective at improving immunisation outcomes due to major system barriers	Unclear risk
18.Implementation of Universal Influenza Immunization Recommendations for Healthy Young Children: Results of a Randomized, Controlled Trial With Registry-Based Recall Kempe et al. 2005 [23]	Prevention of influenza	5193	A regional immunisation registry	The registry contains immunisation data for children in Denver, Colorado, USA	Participant identification and outcome measure detection The intervention group received up to 3 reminder/recall letters, generated by the immunisation registry	Results showed that, in an epidemic influenza year, private practices were able to immunise the majority of 6- to 21-month-old children in a timely manner	Unclear risk
19.The impact of reminder-recall interventions on low vaccination coverage in an inner-city population Le Baron CW et al. 2004 [24]	Low immunisation rates	3050	The MATCH immunisation registry	The MATCH registry contains patient vaccination information within the Atlanta metropolitan area, USA	Participant enrolment (including identification) and outcome measure detection	Large-scale, registry-based reminder-recall interventions produced only small improvements in low immunisation rates of an inner-city population	Unclear risk
20.Identification and recall of children with chronic medical conditions for influenza vaccination Daley et al. 2004 [25]	Prevention of influenza	1851	Immunization Registry Database Denver, Colorado, USA	The registry holds immunisation records for all children < 72 months of age	Participant identification and collection of outcome data was gathered via registry	Diagnosis-based billing data accurately identified children who had HRCs and needed annual influenza vaccination, and registry-driven reminder/recall significantly increased influenza immunisation in targeted children	Unclear risk
21.Effect of four monthly oral vitamin D3 (cholecalciferol) supplementation on fractures and mortality in men and women living in the community: randomised double blind controlled trial Trivedi et al. 2003 [26]	Osteoporotic fractures	2686	British Doctors Study Register General Practice Register	The British doctors study register is a registry based at the Clinical Trials Studies Unit, Oxford The General Practice register is described as the age­sex register of a general practice based in Ipswich, Suffolk	Participant recruitment was via registries. Endpoint ascertainment of mortality via Office for National Statistics	Four monthly supplementations with 100,000 IU oral vitamin D may prevent fractures without adverse effects in men and women living in the general community	Low risk
22. Immunization registry-based recall for a new vaccine Daley et al. 2002 [27]	Prevention of pneumococcal conjugate	1234	Immunization Registry Database	Immunisation registry, Denver, USA	Participant identification and outcome measure via immunisation registry database	Letter and telephone recall for PCV7 vaccine did not significantly increase the rate of PCV7 immunisation in an inner-city teaching hospital serving a disadvantaged population. The effectiveness of recall appears to have been limited by the inability to reach many subjects by mail and telephone	Unclear risk
23.Population-based surveillance by colonoscopy: effect on the incidence of colorectal cancer. Telemark Polyp Study I Thiis – Evensen et al. 1999 [28]	Colorectal cancer	755	Population registry of Telemark, Norway The Norwegian Cancer Registry	Population Registry Cancer Registry	Participant identification and invitation to take part in trial via population registry Outcome detection via Norwegian Cancer Registry	Endoscopic screening examination with polypectomy and follow-up was shown to reduce the incidence of CRC in a Norwegian normal population	Unclear risk
24.Screening for prostate cancer using serum prostate-specific antigen: a randomised, population-based pilot study in Finland Auvinen et al. 1996 [29]	Prostate cancer	230	Finnish Population Registry Finnish Cancer Registry	The Population Registry is a central population registry. The Cancer Registry contains almost complete coverage of cancer diagnosed in Finland	Participant identification via Finnish Population Registry Control follow up via Finnish Cancer Registry	A population-based study in Finland seems feasible and the properties of the PSA test can be regarded as suitable for a randomised screening study	Unclear risk

### *Registry*-l*inked* r*ecruitment*

Nine rRCTs (37.5%) near accomplished or surpassed their recruitment goals [8, 27, 28, 31, 32, 41, 42, 47, 48]. Recruitment with the goal of capturing the largest number of eligible participants available within a registry was present in eight trials (33.3%) [30, 33–38, 40]. Eighteen rRCTs (75%) used registries to identify potential participants for inclusion in a trial [27, 30–42, 44–46]. This included one multicentre rRCT using multiple registries to facilitate participant identification [27].

### *Registry*-l*inked* r*andomisation*

Six rRCTs (25%) randomised participants via a specific randomisation module embedded within the registry [8, 25, 28, 29, 35, 47]. The remaining 18 used randomisation methods external to the registry. Thirteen rRCTs (54.2%) allocated the participants in a 1:1 ratio [8, 25, 27, 28, 31–34, 38–40, 46, 48], for six (25%) the randomisation ratios varied [30, 35, 37, 41, 42, 47] and the remaining five rRCTs (20.8%) did not specify the randomisation ratio [26, 36, 43–45].

### *Registry*-l*inked* o*utcomes*

Twenty-three rRCTs (95.3%) utilised a registry to gather primary/main outcome data. Vaccination rates accounted for 29.2% (*n* = 7) of primary outcome measurements [33–39]. Mortality as a primary outcome was recorded for eight rRCTs (33.3%) [8, 25–28, 40, 42, 48]. Five rRCTs (20.8%) utilized more than one registry to facilitate the acquisition of primary/main outcome measurements [25, 27, 28, 40, 42]. Four rRCTs (25%) utilized the SWEDEHEART Registry in conjunction with pre-existing national healthcare registries to gather outcome/endpoint data [8, 25, 28, 48]. One trial did not include the specifics of linking to a registry; however, evidence from the paper suggests there was record linkage with a nationwide registry [44]. In terms of long-term outcomes, rRCTs gathered data at time points between 5 [26] and 15 years [40].

### *Registry*-l*inked* t*rial* s*pecific* d*ata* c*ollection*

Seven rRCTs (29.2%) used a registry to facilitate trial specific data collection in addition to outcome data [8, 25, 27, 28, 30, 46, 48]. One rRCT requested permission to use data collected until the point of participant ‘opt out’, following intervention [27], and one rRCT continued to collect de-identified data from the registry for ‘non-consenters’ [46].

### *Quality* a*ssurance*

Sixteen rRCTs (66.7%) provided commentary on the quality assurance of registry data [8, 25, 28, 31–39, 41, 44, 45, 48]. Four rRCTs included links to materials which detailed the monitoring of the registry data used in their trials [31, 32, 41, 44]. In two rRCTs (13.3%), investigators assessed the validity of the registry data by comparing the registry data with medical records [35, 39]. Three rRCTs included results of quality assessment evaluations of the registry [36, 38, 45]. The final three rRCTs emphasised the need for improved registry-based quality assurance, following completion of their trials [33, 34, 37]. However, it is not clear if these trials completed study-specific quality assurance checks of the registry data throughout the duration of the trials.

### *Registry*-l*inked* c*ost*-e*ffectiveness*

Just three (12.5%) rRCTs conducted cost-effectiveness assessments [32, 33, 37]. Two trials examined the costs associated with generating reminder/recall notices for infants due/requiring a vaccine [33, 37] and one trial examined the costs associated with the enhancement of an existing UK-based diabetes register [32]. The enhancement of the diabetes registry was a significant initial cost: a total one-off cost of initiating the system across two register areas of UK£27,885 with an additional cost of running the system for the two registers of UK£11,170. The two reminder/recall cost-effectiveness assessments were focused on the effectiveness of these interventions to increase uptake of the vaccine (results showed it was age-dependant), rather than the cost-effectiveness of the use of the registry to conduct the trial.

### *Registry*-l*inked* i*nterventions*

For nine rRCTs (35.5%), a registry was used by researchers to facilitate either the development or delivery of trial interventions to participants [31, 33–38, 41, 47]. These were four (44.4%) immunisation reminder/recall trials whereby a registry generated the reminder/recall interventions [33–36]; one (11.1%) additional immunisation reminder/recall trial where researchers determined the requirement for an additional immunisation reminder based on a participants immunisation status in the registry [38]; one (11.1%) cancer screening trial where the intervention group were contacted prior to receiving an intervention kit, and given the opportunity to either cancel the intervention or update their personal registry information, thus amending their eligibility in the trial [41]; one (11.1%) smoking cessation trial in which participants received an allocated intervention when they logged onto the registry [47]; and for two trials (22.2%), the precise details of how the registry facilitated the application of the intervention were not discussed [31, 37].

### *Informed* c*onsent*

Of the 24 rRCTs, 7 (29.2%) sought informed consent prior to randomisation [8, 25, 26, 28, 30, 31, 48] and 2 (8.3%) completed randomisation before consent [46, 47]. For two trials (8.3%), the ethical approvals differed by jurisdiction, with some granting a waiver of consent and others requiring an opt out approach [27, 32]. Five trials (20.8%) randomised participants to screening or control groups without informed consent [40, 42–45]; however, three trials subsequently requested consent from the screening cohort [40, 42, 44]. It was not specified if informed consent was required or sought for the remaining two (8.3%) rRCTs [43, 45]. Six additional rRCTs (25%) randomised minors without acquiring parental consent [33, 34, 36–39]. Parents were subsequently contacted following randomisation in relation to their child’s vaccination status, but it is not stated if consent was then required. Two trials were granted waiver/exemption status [35, 41].

### Risk of *bias*

Risk of bias assessments and the reasons for the judgements for the overall risk of bias result for the 24 rRCTs are available in supplementary file 3. Overall, the authors judged 5 trials (20.8%) to have a high risk of bias, 17 trials (70.8%) to have an unclear risk of bias and 2 trials (8.3%) to have a low risk of bias. The judgement for all high risk of bias trials was consistently due to performance bias (blinding of participants and personnel domain) and detection bias (blinding of outcome assessment domain). Additional to a high risk of both performance and detection bias, Young et al. [27] had a high-risk judgement for selection bias (allocation concealment domain). 

## Discussion

Our systematic review included 24 rRCTs that utilised a patient registry to facilitate both the participant recruitment process and the collection of outcome data. We found that the interpretation of a registry was diverse. This is reflected in the variation of how investigators used registries, and in their reporting (Table 1). We find the advantages of rRCTs are recruitment efficiency, shorter trial times, cost effectiveness, outcome data completeness, smaller carbon footprint, lower participant burden and ability to conduct multiple trials from the same registry. Challenges are data collection/management, quality assurance issues and the timing of informed consent.

A minority of trials (*n* = 4) utilised a registry to enable the majority of key trial processes; recruitment, randomisation, trial specific data collection and the collection of trial outcomes/end points [8, 25, 28, 48]. These trials surpassed their sample size requirements, had almost complete follow-up, reported minimal missing data and were performed at a relatively low cost. All were conducted in the SWEDEHEART registry [49]. SWEDEHEART has many advantages as it was designed to facilitate the conduct of clinical research and clinical trials. SWEDEHEART is continuously monitored for data quality and education and training for users of the registry is provided [50]. It is a good exemplar of quality assurance systems in rRCTs. However, validation of the population registries used to collect additional outcome data in conjunction with SWEDEHEART is not discussed and warrants further attention to comply with the SWEDEHEART quality assurance standards.

Many rRCTs surpassed their sample size requirements [8, 25, 27, 28, 40, 42, 47, 48]. Whilst it is widely documented that a considerable proportion of RCTs fail due to recruitment issues [51, 52], rRCTs do not have the same issue and allow for a more efficient recruitment process. One issue that has not been resolved is that of informed consent when conducting rRCTs and our results show this is variable, with some waiving consent, and others taking consent either before or after randomisation.

The collection of trial data can consume vast proportions of trial resources and significantly increase trial costs [53] as well as increase the carbon footprint [54]. Utilising a web-based approach to capture trial data within a registry can significantly reduce trial costs and minimise a trial’s carbon footprint [54] and preserve the environment for future generations.

There are interesting lessons to be learned from colleagues who have repeatedly and successfully conducted rRCTs. For example, Bohlin et al. [47] used the GynOp register in a near identical fashion to their Swedish colleagues in SWEDEHEART and reported similar efficiency results in terms of high recruitment rates, minimal missing data and low costs [47]. These rRCTs investigated wholly different conditions, but by applying almost identical registry-based methodology, successfully combined high recruitment with low cost. Whilst authors cite the low costs, a cost-effectiveness study off rRCTs compared to a traditional RCT is not included in the cost-effectiveness examples given in this study. There are initial set-up costs which are considerable, as per the example of the diabetes registry given in the section, " Registry-linked cost-effectiveness" [32]. We speculate that the suitability and cost-effectiveness of registries for conducting trials will vary, with the most suitable and cost-effective being those established with the intention to embed clinical trials. Trials from the GynOp and SWEDEHEART registries are prime examples of the potential of rRCTs, when using established registries for the majority, or all, key trial procedures. It is also possible for rRCTs to facilitate the development and delivery of a range of trial interventions [31, 33–38, 41, 47]. The diverse use of registries within trials is a clear strength of registry-based methodology.

The ability to complete randomisation blinded within a registry is unique and worth noting. Six rRCTs in our study had a randomisation module embedded within the registry [8, 25, 28, 35, 47, 48]. Randomisation is the only definitive technique to control for confounding factors within trial groups [6]. The benefits of having an embedded randomisation module within a registry include an automated, effective enrolment process with a minimally selected cohort of patients [6, 55]. We reviewed some protocols and note that the inclusion of an embedded randomisation module is becoming more frequent [17, 56–58]. It is particularly useful when randomisation is time sensitive.

Outcome data collection was the most common trial procedure facilitated by a registry. The collection of certain outcomes, e.g. mortality, can be gathered consistently across various types of registries [59]. The advantages of gathering trial outcome data via a registry include a significant reduction in trial costs [1, 60], minimisation of study visits facilitating a lower participant burden, the potential to capture almost all trial follow-up data which reduces staff burden [7] and a reduced carbon footprint for the trial. In one trial, long-term follow-up data was retrieved from a registry 15 years later [40]. The burden of obtaining long-term participant data within RCTs include both logistical and financial constraints [61]. Provided researchers are confident in the quality of the data they are collecting, the use of registries can be advocated for the collection of long-term outcome data.

Concerns have been raised about the quality and completeness of registry data [7, 62] and this remains a significant challenge for rRCTs. Trials support decisions through the data they collect. Even for traditional RCTs, the quality of the data is key to ensuring the trial supports better and more informed decisions and meets its aim. If we cannot trust the data, the trial has failed. Errors in the data collection process not only affect the safety of the patients in the trial through the introduction of bias but also affect the safety of future patients. rRCTs pose a unique challenge because in many cases, registries are not designed with trial conduct in mind. Thus, the trial can be limited to collecting the outcome data as presented in the registry, regardless of its completeness or suitability. Data may also be missing, or the data entry might occur long after the data collection; hence, it may not suit the trial timeline. Across registries, terminology may not be consistent. Key to advancing rRCT conduct will be standardising data collection across registries to follow international health data terminology standards and definitions, and improving data linkage. This will enhance analytical capabilities, making clinical trials more cost-effective and improving the comprehensiveness of post-market surveillance for devices and medicines. We found a large variation in the reporting of quality assurance of the registry data used in rRCTs. Only 16 discussed validation of registry data and 3 of those expressed the need for additional validation of registry data, following completion of their trials. We recommend that new trials that include data from registries implement rigorous quality assurance systems at the trial design stage.

For many trials (70.8%), the overall risk of bias judgement was unclear as there was insufficient information provided to state, with certainty, if a trial was at a high or low risk of bias. rRCTs replicate real life, given that participants are only told about an intervention when they are going to receive it and not if they are not to receive it. In this respect, they are considered pragmatic. However, it is also argued that rRCTs generate an artificial environment, given that they allow for longer follow-up periods than traditional RCTs which arguably affects the external validity [63]. The discussion on bias in rRCTs is limited in the literature. Whilst the completeness of the data in rRCTs can reduce attrition bias, the risk of residual bias when trying to understand causation is high. We suggest bias in rRCTs is under researched and should be considered by methodologists and statisticians and an appropriate guidance/manuscript document developed.

Another matter requiring discussion is the ethics on the timing and conditions of informed consent. We found this was variable between rRCTs and dependent on the ethical approval conditions. Some rRCTs opted for the ‘Zelen design’ approach [64], randomising participants prior to consent [40, 42, 44, 46, 47]. Others took oral consent, randomised the participants and then followed up with written consent. For many trials, it was not clear when consent was obtained [33, 34, 36–39, 43, 45]. In some circumstances, a waiver of consent was granted [27, 35, 41]. A review of the ethical issues of informed consent in rRCTs is warranted.

The nomenclature used to describe rRCTs is inconsistent and is a significant barrier to their use in the long-term. It creates difficulties for systematic reviews and meta-analyses, as we have found here. Many rRCTs did not integrate the term ‘registry-based’ in their title; however, some did incorporate the terminology within their protocol publication [8, 27, 28]. rRCTs can currently be divided into two groups: trials that are deeply embedded in registries and utilise the registry to facilitate most, if not all, key trial processes, e.g. recruitment, randomisation, outcome data collection; and trials that simply utilise a registry to facilitate one specific function, e.g. outcome data collection. However, where the use of a registry is limited to a singular function; it is questionable if these trials should be classified as rRCTs. Given the variability, there remains an urgent need for consensus on a definition for an rRCT. RCTs are intervention studies and are so-called because the investigator intervenes. In our view, investigators in rRCTs can only intervene in the allocation and the timing of the intervention. They do not have control over the outcome data (but can select the outcome based on the data available in the registry). This might be a useful start to thinking about defining rRCTs.

### *Strengths and* l*imitations*

This study is a comprehensive systematic review and includes a risk of bias assessment for each of the included trials. During the data extraction process, it was often necessary to expand our search to protocol papers or registry citations to comprehensively extract and understand the classification of registry use. We found that the reporting of how a registry was used was limited and variable from study to study. This also means that we have potentially missed some studies for inclusion in our review, but we have no means of identifying that. We chose to limit our inclusion to registry-based studies that included both recruitment of participants and outcome collection, as we are interested in furthering the literature on rRCTs of this nature, which we believe offer significant advantages to facilitating the conduct of trials as part of routine clinical care. We acknowledge that we have thus missed out on some studies that used a registry for recruitment only, or used a registry for outcome collection only, but these would not contribute to the purpose of our systematic review.

## Conclusion

The results of this study highlight the fact that design of rRCTs is bespoke and dependent on the capabilities of the registry. Even within the rRCTs, we have also established the variability evident in many of the processes: ethics and consent, randomisation, data collection, outcome data and trial reporting. The advantages to rRCTs include recruitment efficiency and shorter trial times, cost-effectiveness, outcome data completeness, a smaller carbon footprint, lower participant burden and the ability to conduct multiple trials using the same registry. The challenges to rRCTs are data collection and management, limitation of outcome measures, quality assurance issues and the timing and ethics of informed consent.

The cornerstone of any functioning health care system is quality research. The quality of the data collection is key to ensuring the trial supports better and more informed decisions and meets its aim. If we cannot trust the data, the trial has failed. We welcome the CONSORT extension for the reporting of randomised controlled trials conducted using cohorts and routinely collected data [13]. This will be crucial to allow trialists to clearly think about the outcomes at the design phase. We suggest the inclusion of the term ‘registry-based’ in the trial title of all RCTs utilising a registry and the clear and simple breakdown of the registry-based conduct of the trial in the abstract to allow indexing in the major databases. The issue of bias in rRCTs is under researched and reported and discussion of this in the literature would be welcomed as a matter of priority. Researchers should endeavour to maximise the use of a registry where feasible; however, it is critical that the quality assurance of all registry data is given key consideration at the trial design stage.

### Supplementary Information

Supplementary Material 1.

Supplementary Material 2.

Supplementary Material 3.

## Data Availability

All data generated or analysed during this study are included in this published article [and its supplementary information files].
