# Supplementary material for: Registry-based randomised controlled trials: conduct, advantages and challenges—a systematic review
Source: Trials. 2024 Jun 11;25:375. doi: 10.1186/s13063-024-08209-3 (PMC11165819; doi:10.1186/s13063-024-08209-3)
Supplement: Supplementary file 1 — Supplementary Material 1. [file 13063_2024_8209_MOESM1_ESM.docx]

Search String Results from databases:

Database 1. PUBMED

randomised OR randomized OR randomized controlled trial OR randomized controlled trial (MESH) OR RCT OR “randomized clinical trial” OR pragmatic trial OR randomized database trial OR randomized registry trial OR “database study”

AND

Registry (MESH) OR “registry based” OR registry based OR register based OR “registry trial” OR rRCT OR register

Search per PUBMED: (((((((((randomised[Title/Abstract] OR randomized[Title/Abstract]) OR randomized controlled trial[Title/Abstract]) OR "Randomized Controlled Trials as Topic"[Mesh]) OR RCT[Title/Abstract]) OR "randomized clinical trial"[Title/Abstract]) OR pragmatic trial[Title/Abstract]) OR (("random allocation"[MeSH Terms] OR ("random"[All Fields] AND "allocation"[All Fields]) OR "random allocation"[All Fields] OR "randomized"[All Fields]) AND database trial[Title/Abstract])) OR randomized registry trial[Title/Abstract]) OR "database study"[Title/Abstract]) AND (((((("Registries"[Mesh] OR "registry based"[Title/Abstract]) OR registry based[Title/Abstract]) OR register based[Title/Abstract]) OR "registry trial"[Title/Abstract]) OR rRCT[Title/Abstract]) OR register[Title/Abstract])

Database 2. Embase (OVID Interface)

randomised:ab,ti OR randomized:ab,ti OR ('randomized controlled trial':ab,ti AND topic:ab,ti) OR rct:ab,ti OR 'pragmatic trial':ab,ti OR 'randomized database trial':ab,ti OR 'randomized clinical trial':ab,ti OR 'randomized registry trial':ab,ti OR 'database study':ab,ti

AND

registry:ab,ti OR 'registry based':ab,ti OR 'register based':ab,ti OR 'registry trial':ab,ti OR rrct:ab,ti OR register:ab,ti

Database 3. Cinahl Plus

randomised controlled trial or randomized controlled trial or rct (ABSTRACT) OR randomized controlled trial (as SUBJECT) OR “randomized clinical trial”(ABSTRACT) OR pragmatic trials (ABSTRACT) OR randomized database trial (ABSTRACT) OR randomized registry trial (ABSTRACT) OR “database study” (ABSTRACT)

AND

Registry (SUBJECT) OR register-based study (ABSTRACT) OR registry based (ABSTRACT) OR registry study (ABSTRACT) OR rRCT (ABSTRACT) OR register (ABSTRACT)

((SU registry OR AB register-based study OR AB registry based OR AB registry study OR AB rRCT OR AB register) AND (S1)) AND (S1 AND S2)

Database 4: SCOPUS

TITLE-ABS-KEY (randomised OR randomized OR randomized AND controlled AND trial OR "randomized controlled trial" OR rct OR "randomized clinical trial" OR pragmatic AND trial OR "randomized database trial" OR "randomized registry trial" OR "database study" ) AND TITLE-ABS-KEY ( registry OR "registry based" OR registry AND based OR register AND based OR "registry trial" OR rrct OR register ) )

Database 5: Cochrane Controlled Register of Trials (CENTRAL)

Title/Abstract/Keyword:

"randomised clinical trial" OR "randomized control trial" OR randomised OR randomized OR RCT pragmatic trial OR randomized database trial OR randomized registry trial OR “database study”AND

Registry OR “registry based” OR registry based OR register based OR “registry trial” OR rRCT OR register
