# Supplementary material for: Registry-based randomised controlled trials: conduct, advantages and challenges—a systematic review
Source: Trials. 2024 Jun 11;25:375. doi: 10.1186/s13063-024-08209-3 (PMC11165819; doi:10.1186/s13063-024-08209-3)
Supplement: Supplementary file 3 — Supplementary Material 3. [file 13063_2024_8209_MOESM3_ESM.docx]

**Supplementary File 3: Risk of Bias (Cochrane Risk of Bias Tool (22))**

| Author | Selection bias | | Performance  Bias | Detection  Bias | Attrition  Bias | Reporting  Bias | Other  Bias | Overall Risk of  Bias* |
| --- | --- | --- | --- | --- | --- | --- | --- | --- |
|  | Random  sequence  generation | Allocation  concealment | Blinding of  participants and personnel | Blinding of  outcome  assessment | Incomplete  outcome  data | Selective  outcome  reporting | Other  sources  of bias | Overall  risk of  bias |
| Young et al. 2020^(1)^  Bohlin et al.2020^(2)^  Holme et al.2018^(3)^  Hofmann et al.^(4)^  Gotberg et al.^(5)^  Navaneethan et al.^(6)^  Erlinge at al.^(7)^  Sultana et al.^(8)^  Bretthauer et al.^(9)^  Chamany et al.^(10)^  Dombkowski et al.^(11)^  Frobert et al.^(12)^  Hall et al.^(13)^  Dombkowski et al.^(14)^  Malila et al. ^(15)^  Eccles et al.^(16)^  Irigoyen et al.^(17)^  Kempe et al.^(18)^  Lebaron et al.^(19)^  Daley et al.^(20)^  Trivedi et al.^(21)^  Daley et al.^(22)^  Thiis Evensen et al.^(23)^  Auvinen et al.^(24)^ | Low risk High risk  Low risk Unclear risk  Low risk Unclear risk  Low risk Low risk  Low risk Unclear risk  Low risk Unclear risk  Low risk Unclear risk  Low risk Unclear risk  Unclear risk Unclear risk  Low risk Low risk  Unclear risk Unclear risk  Low risk Unclear risk  Unclear risk Unclear risk  Unclear risk Unclear risk  Unclear risk Unclear risk  Low risk Low risk  Low risk Unclear risk  Low risk Unclear risk  Low risk Unclear risk  Low risk Unclear risk  Unclear risk Unclear risk  Low risk Unclear risk  Unclear risk Unclear risk  Unclear risk Unclear risk | | High risk  Low risk  Low risk  High risk  High risk  High risk  High risk  High risk  Unclear risk  Unclear risk  Unclear risk  High risk  Unclear risk  Unclear risk  High risk  Unclear risk  High risk  Unclear risk  High risk  Unclear risk  Low risk  Low risk  Unclear risk  Unclear risk | High risk  Unclear risk  Low risk  High risk  High risk  Low risk  High risk  High risk  Unclear risk  Unclear risk  Unclear risk  High risk  Unclear risk  Unclear risk  Unclear risk  Unclear risk  High risk  Unclear risk  High risk  Unclear risk  Low risk  Unclear risk  Unclear risk  Unclear risk | Low risk  Low risk  Low risk  Low risk  Low risk  Low risk  Low risk  Low risk  Unclear risk  Low risk  Low risk  Low risk  Low risk  Low risk  Low risk  Low risk  Low risk  Low risk  Low risk  Low risk  Low risk  Low risk  Low risk  Low risk | Low risk  Low risk  Low risk  Low risk  Low risk  Low risk  Low risk  Low risk  Low risk  Low risk  Low risk  Low risk  Low risk  Low risk  Low risk  Low risk  Low risk  Low risk  Low risk  Low risk  Low risk  Low risk  Low risk  Low risk | Low risk  Unclear risk  Low risk  Low risk  Low risk  Low risk  Low risk  High risk  High risk  Unclear risk  Unclear risk  High risk  High risk  High risk  Low risk  Unclear risk  Unclear risk  Unclear risk  Unclear risk  Unclear risk  Unclear risk  Unclear risk  Unclear risk  Unclear risk | High risk  Unclear risk  Low risk  High risk  High risk  Unclear risk  High risk  Unclear risk  Unclear risk  Unclear risk  Unclear risk  High risk  Unclear risk  Unclear risk  Unclear risk  Unclear risk  Unclear risk  Unclear risk  Unclear risk  Unclear risk  Low risk  Unclear risk  Unclear risk  Unclear risk |

**Overall risk of bias judgement criteria (25):*

*Low risk of bias: Most information is from trials at low risk of bias.*

*Unclear risk of bias: Most information is from trials at low or unclear risk of bias.*

*High risk of bias: The proportion of information from trials at a high risk of bias is sufficient to affect the interpretation of results*

**Justification for Overall Risk of Bias Assessment**

| Author Name | Overall Risk of Bias Judgement | Justification for Overall Risk of Bias Result |
| --- | --- | --- |
| Young et al. 2020 | High risk | Potential for a high risk of bias due to trial personnel and outcome assessors being aware of treatment allocation. |
| Bohlin et al. 2020 | Unclear risk | It is not possible to determine if this trial is at a low risk of bias as allocation concealment is unclear, blinding of outcome assessment is unclear and additional bias may exist such as recall and response bias (questionnaire based research). We do not deem the trial to be at a high risk of bias. |
| Holme et al. 2018 | Low risk | This trial is at a low risk of bias as most key domains are at a low risk of bias. |
| Hoffmann et al. 2017 | High risk | Potential for a high risk of bias due to trial personnel and outcome assessors being aware of treatment allocation. |
| Gotberg et al. 2017 | High risk | Potential for a high risk of bias due to trial personnel and outcome assessors being aware of treatment allocation. |
| Navaneethan et al. 2017 | Unclear risk | This trial is deemed unclear risk of bias as we are not sure to the extent that performance bias (which is high risk) could influence the results of the trial. |
| Erlinge et al. 2017 | High risk | Potential for a high risk of bias due to trial personnel and outcome assessors being aware of treatment allocation. |
| Sultana et al. 2016 | Unclear risk | This trial is deemed unclear risk of bias as we are unsure to the extent performance bias and detection bias (both high risk) would impact the results of the primary outcome of the trial (trial participation as indicated by returning a swab or undergoing a Pap test). |
| Bretthauer et al. 2016 | Unclear risk | This trial is deemed unclear risk of bias as there is not enough information in the paper for most domains to make a high or low judgement, including an overall risk of bias judgement. |
| Chamany et al. 2015 | Unclear risk | This trial is deemed unclear risk of bias as we are not sure to the extent that performance bias and detection bias (which are unclear for risk of bias) could influence the results of the trial. |
| Dombkowski et al. 2014 | Unclear risk | This trial is deemed unclear risk of bias as there is not enough information in the paper for most domains to make a high or low judgement, including an overall risk of bias judgement. |
| Frobert et al. 2013 | High risk | Potential for a high risk of bias due to trial personnel and outcome assessors being aware of treatment allocation. |
| Hall et al. 2012 | Unclear risk | This trial is deemed unclear risk of bias as there is not enough information in the paper for most domains to make a high or low judgement, including an overall risk of bias judgement. |
| Dombkowski et al. 2012 | Unclear risk | This trial is deemed unclear risk of bias as there is not enough information in the paper for most domains to make a high or low judgement, including an overall risk of bias judgement. |
| Malila et al. 2008 | Unclear risk | This trial is deemed unclear risk of bias as the following domains are unclear for risk of bias: selection bias, detection bias and other bias. Performance bias is deemed high risk however this is relation to the trial participants, it is unclear is trial personnel are blinded. It is not possible to determine if this trial is high or low risk of bias based on these domains. |
| Eccles et al. 2007 | Unclear risk | This trial is deemed unclear as there is not enough information in the paper in relation to performance bias and detection bias to make a high or low judgement, while selection bias and attrition bias and reporting are bias are low, we do not know if the unclear domains could bias the outcomes of the study (the study outcomes were the clinical process and outcome variables held on the diabetes register, patient-reported outcomes, and service and patient costs). |
| Irigoyn et al. 2006 | Unclear risk | This trial is deemed unclear risk of bias as we are unsure to the extent performance bias and detection bias (both high risk) would impact the results of the primary outcome of the trial (2 outcomes – received subsequent immunisation and age appropriate up to date status) |
| Kempe et al. 2005 | Unclear risk | This trial is deemed unclear risk of bias as there is not enough information in the paper for most domains to make a high or low judgement, including an overall risk of bias judgement. |
| Le Baron et al. 2004 | Unclear risk | This trial is deemed unclear risk as it is not possible to determine if the potential for performance bias and detection bias (both high risk) would significantly impact the outcome (4313 series completion by 24 months of age for valid vaccination doses) |
| Daley et al. 2004 | Unclear risk | This trial is deemed unclear risk as it is not possible to determine if the potential for selection bias, performance bias, detection bias and other bias (all unclear risk) would significantly impact the outcome (receipt of vaccine) |
| Trivedi et al. 2003 | Low risk | This trial is deemed low risk as most domains are low risk and we do not think the unclear risk domains would impact the outcomes of the study (fracture incidence and total mortality by cause) |
| Daley et al. 2002 | Unclear risk | This trial is deemed unclear risk as it is not possible to determine if the potential for selection bias, detection bias and other bias (all unclear risk) would significantly impact the outcome (receipt of vaccine during study period as recorded in the registry) |
| Thiis-Evensen et al. 1999 | Unclear risk | This trial is deemed unclear risk of bias as there is not enough information in the paper for most domains to make a high or low judgement, including an overall risk of bias judgement. |
| Auvinen et al. 1996 | Unclear risk | This trial is deemed unclear risk of bias as there is not enough information in the paper for most domains to make a high or low judgement, including an overall risk of bias judgement. |

1. Young PJ, Bagshaw SM, Forbes AB, Nichol AD, Wright SE, Bailey M, et al. Effect of Stress Ulcer Prophylaxis With Proton Pump Inhibitors vs Histamine-2 Receptor Blockers on In-Hospital Mortality Among ICU Patients Receiving Invasive Mechanical Ventilation: The PEPTIC Randomized Clinical Trial. Jama. 2020;323(7):616-26.

2. Bohlin KS, Löfgren M, Lindkvist H, Milsom I. Smoking cessation prior to gynecological surgery-A registry-based randomized trial. Acta Obstet Gynecol Scand. 2020;99(9):1230-7.

3. Holme Ø, Løberg M, Kalager M, Bretthauer M, Hernán MA, Aas E, et al. Long-Term Effectiveness of Sigmoidoscopy Screening on Colorectal Cancer Incidence and Mortality in Women and Men: A Randomized Trial. Ann Intern Med. 2018;168(11):775-82.

4. Hofmann R, James SK, Jernberg T, Lindahl B, Erlinge D, Witt N, et al. Oxygen Therapy in Suspected Acute Myocardial Infarction. N Engl J Med. 2017;377(13):1240-9.

5. Götberg M, Christiansen EH, Gudmundsdottir IJ, Sandhall L, Danielewicz M, Jakobsen L, et al. Instantaneous Wave-free Ratio versus Fractional Flow Reserve to Guide PCI. N Engl J Med. 2017;376(19):1813-23.

6. Navaneethan SD, Jolly SE, Schold JD, Arrigain S, Nakhoul G, Konig V, et al. Pragmatic Randomized, Controlled Trial of Patient Navigators and Enhanced Personal Health Records in CKD. Clin J Am Soc Nephrol. 2017;12(9):1418-27.

7. Erlinge D, Omerovic E, Fröbert O, Linder R, Danielewicz M, Hamid M, et al. Bivalirudin versus Heparin Monotherapy in Myocardial Infarction. N Engl J Med. 2017;377(12):1132-42.

8. Sultana F, English DR, Simpson JA, Drennan KT, Mullins R, Brotherton JM, et al. Home-based HPV self-sampling improves participation by never-screened and under-screened women: Results from a large randomized trial (iPap) in Australia. Int J Cancer. 2016;139(2):281-90.

9. Bretthauer M, Kaminski MF, Løberg M, Zauber AG, Regula J, Kuipers EJ, et al. Population-Based Colonoscopy Screening for Colorectal Cancer: A Randomized Clinical Trial. JAMA Intern Med. 2016;176(7):894-902.

10. Chamany S, Walker EA, Schechter CB, Gonzalez JS, Davis NJ, Ortega FM, et al. Telephone Intervention to Improve Diabetes Control: A Randomized Trial in the New York City A1c Registry. Am J Prev Med. 2015;49(6):832-41.

11. Dombkowski KJ, Costello LE, Harrington LB, Dong S, Kolasa M, Clark SJ. Age-specific strategies for immunization reminders and recalls: a registry-based randomized trial. Am J Prev Med. 2014;47(1):1-8.

12. Fröbert O, Lagerqvist B, Olivecrona GK, Omerovic E, Gudnason T, Maeng M, et al. Thrombus aspiration during ST-segment elevation myocardial infarction. N Engl J Med. 2013;369(17):1587-97.

13. Hall AE, Sanson-Fisher RW, Lynagh MC, Threlfall T, D'Este CA. Format and readability of an enhanced invitation letter did not affect participation rates in a cancer registry-based study: a randomized controlled trial. J Clin Epidemiol. 2013;66(1):85-94.

14. Dombkowski KJ, Harrington LB, Dong S, Clark SJ. Seasonal influenza vaccination reminders for children with high-risk conditions: a registry-based randomized trial. Am J Prev Med. 2012;42(1):71-5.

15. Malila N, Oivanen T, Malminiemi O, Hakama M. Test, episode, and programme sensitivities of screening for colorectal cancer as a public health policy in Finland: experimental design. Bmj. 2008;337:a2261.

16. Eccles MP, Whitty PM, Speed C, Steen IN, Vanoli A, Hawthorne GC, et al. A pragmatic cluster randomised controlled trial of a Diabetes REcall And Management system: the DREAM trial. Implement Sci. 2007;2:6.

17. Irigoyen MM, Findley S, Wang D, Chen S, Chimkin F, Pena O, et al. Challenges and successes of immunization registry reminders at inner-city practices. Ambul Pediatr. 2006;6(2):100-4.

18. Kempe A, Daley MF, Barrow J, Allred N, Hester N, Beaty BL, et al. Implementation of universal influenza immunization recommendations for healthy young children: results of a randomized, controlled trial with registry-based recall. Pediatrics. 2005;115(1):146-54.

19. LeBaron CW, Starnes DM, Rask KJ. The impact of reminder-recall interventions on low vaccination coverage in an inner-city population. Arch Pediatr Adolesc Med. 2004;158(3):255-61.

20. Daley MF, Barrow J, Pearson K, Crane LA, Gao D, Stevenson JM, et al. Identification and recall of children with chronic medical conditions for influenza vaccination. Pediatrics. 2004;113(1 Pt 1):e26-33.

21. Trivedi DP, Doll R, Khaw KT. Effect of four monthly oral vitamin D3 (cholecalciferol) supplementation on fractures and mortality in men and women living in the community: randomised double blind controlled trial. Bmj. 2003;326(7387):469.

22. Daley MF, Steiner JF, Brayden RM, Xu S, Morrison S, Kempe A. Immunization registry-based recall for a new vaccine. Ambul Pediatr. 2002;2(6):438-43.

23. Thiis-Evensen E, Hoff GS, Sauar J, Langmark F, Majak BM, Vatn MH. Population-based surveillance by colonoscopy: effect on the incidence of colorectal cancer. Telemark Polyp Study I. Scand J Gastroenterol. 1999;34(4):414-20.

24. Auvinen A, Tammela T, Stenman UH, Uusi-Erkkilä I, Leinonen J, Schröder FH, et al. Screening for prostate cancer using serum prostate-specific antigen: a randomised, population-based pilot study in Finland. Br J Cancer. 1996;74(4):568-72.

25. Higgins JP, Altman DG, Gøtzsche PC, Jüni P, Moher D, Oxman AD, et al. The Cochrane Collaboration's tool for assessing risk of bias in randomised trials. Bmj. 2011;343:d5928.
